# Performance of exercise transcutaneous oximetry versus imaging at the buttock, thigh and calf level for the diagnosis of peripheral artery disease

**DOI:** 10.1111/cpf.70068

**Published:** 2026-05-18

**Authors:** Mariève Houle, Simon Lecoq, Mathieu Feuilloy, Jeanne Hersant, Samir Henni, Pierre Abraham

**Affiliations:** ^1^ Université d′Angers, Inserm, CNRS, MITOVASC, Equipe CarMe, SFR ICAT Angers France; ^2^ Service of Sports Medicine Angers University Hospital Angers France; ^3^ Service of Vascular Medicine Angers University Hospital Angers France; ^4^ School of Electronics (ESEO) Angers France; ^5^ UMR CNRS Le Mans France

**Keywords:** accuracy, diagnosis, lower extremity artery disease, lower limb ischemia, treadmill, validation, walking test

## Abstract

**Background:**

Exercise transcutaneous oximetry (Ex‐TcPO_2_), through the minimal value of the “decrease from rest of oxygen pressure” (DROPmin), identifies ischemia during exercise in patients with PAD. However, its diagnostic performance at the thigh level compared to imaging modalities, has not been evaluated. The objective of this retrospective study was to perform an external validation of Ex‐TcPO₂ results at the buttock and calf, a first validation of Ex‐TcPO₂ at the thigh level and to determine the respective diagnostic performance of the DROPmin and DROPend (the DROP at the time of exercise cessation).

**Methods:**

We studied 192 patients complaining of intermittent claudication. We retrieved DROPmin and DROPend values from Ex‐TcPO_2_ testing and the presence/absence of an ipsilateral significant stenosis ( ≥ 70%) at imaging.

**Results:**

The performance of Ex‐TcPO₂ using DROPmin values at the buttock, thigh and calf levels were respectively fair (AUC = 0.70; 95% CI: 0.64–0.76), fair (AUC = 0.77; 95% CI: 0.72–0.83) and excellent (AUC = 0.93; 95% CI: 0.90–0.96). When using DROPend, the performance of Ex‐TcPO_2_ was no more significant.

**Discussion/Conclusion:**

This study showed that Ex‐TcPO₂ has a fair to excellent accuracy for detecting the presence of at least one artery stenosis ≥70% in the lower limb according to buttock, thigh and calf area in highly selected patients. This study also highlights the importance of using the minimal value of DROPmin rather than the DROPend.

## 1 INTRODUCTION

Peripheral artery disease (PAD) is a chronic condition commonly associated with atherosclerotic narrowing of the arteries (Nordanstig et al., 2024), particularly in the lower limbs with clinical repercussion in regions such as the buttocks, thighs, and calves (Aday & Matsushita, 2021). In 2015, the Global Peripheral Artery Disease Study estimated the global prevalence of PAD at approximately 237 million individuals (Song et al., 2019). Among the risk factors for PAD, the most common include advanced age, diabetes, smoking, obesity, hypertension, and a history of concomitant cardiovascular disease (Abramson et al., 2022; Hamburg & Creager, 2017; Hossain et al., 2019; Kim et al., 2023; Song et al., 2019). In individuals with symptomatic PAD, the most common clinical manifestation is vascular intermittent claudication (Fowkes et al., 2017; McDermott et al., 2013) which is characterized by lower limb pain or discomfort that worsens with physical activities, such as walking, and typically relieved by rest within 10 min (Abramson et al., 2022; McDermott et al., 2013; Nordanstig et al., 2024; Rose, 1962). The presence of leg symptoms such as vascular intermittent claudication generally results from insufficient oxygen delivery to active muscles (Hossain et al., 2019).

To date, the diagnosis of PAD is generally conducted using the ankle‐brachial index (ABI) at rest with a score ≤ 0.90 suggesting the presence of PAD (Arain et al., 2012; Nordanstig et al., 2024). However, the ABI does not define the level of the lesion and can be altered in several chronic conditions such as diabetes (Aboyans et al., 2012; Kim et al., 2023; Potier et al., 2011) or kidney disease (Garimella & Hirsch, 2014), as well as in older individuals (McDermott et al., 2013) or in those with PAD that only becomes apparent after exercise (Fowkes et al., 2017). For that reason, other non‐invasive tests such as imaging techniques including duplex ultrasound (DUS) and computed tomography angiography (CTA) and magnetic resonance angiography (MRA) are regularly prescribed to confirm the presence of arterial lesions in the lower limbs (Nordanstig et al., 2024). Although radiological techniques are recognized as reference evaluations, they remain expensive tools (Kamenský et al., 2024). In addition, these techniques are conducted at rest while vascular intermittent claudication associated with PAD occurs during exercise. Further, due to the presence of collateral vessels, the presence of arterial lesions is not a proof of the presence of underlying ischemia during exercise. Thereby and logically, any technique aiming to evaluate exercise‐related ischemia using imaging as a gold standard, if accurate, is expected to show a high specificity but shall inevitably face apparent low sensitivity.

Since several years, exercise transcutaneous oximetry (Ex‐TcPO₂) has been introduced as a non‐invasive test in the clinical evaluation of PAD. In fact, the technique has grown in interest in the medical domain (Abraham et al., 2018; Abraham et al., 2021; Mahé et al., 2021). Ex‐TcPO₂ allows the measurement of the partial pressure of oxygen that diffuses from subcutaneous tissue to the skin surface while the patient is simultaneously walking on a treadmill following a standardized walking test. Although a surface technique, the Ex‐TcPO₂ is an interesting tool, as it can provide clinically relevant information regarding the presence of regional ischemia as a cause of walking limitations in patients with suspected diagnosis of PAD (Jaquinandi et al., 2007). By capturing exercise‐induced ischemia that may not be detectable through resting measurement, the Ex‐TcPO_2_ provides functional insights about the PAD condition that support both diagnosis and therapeutic decision‐making. A previous study showed that, when using a decrease from rest of oxygen pressure (DROP) value of −15 mmHg, the Ex‐TcPO₂ technique yielded ROC curves indicating a sensitivity of 82.8% and a specificity of 82.1% for the detection of proximal (buttock) arterial ischemia, based on the comparison between arteriography and TcPO₂ values (Abraham et al., 2003). Furthermore, comparison of the Ex‐TcPO₂ technique with CTA for the detection of both proximal (buttock) and distal (calf) ischemia confirmed its comparable diagnostic accuracy, reinforcing its validity as a non‐invasive assessment technique (Audonnet et al., 2017; Koch et al., 2016). However, the diagnostic performance of Ex‐TcPO₂ in detecting arterial stenosis, with imaging modalities as the reference standard especially at the thigh level has not been evaluated. Since an exercise‐induced hypoxemia and contralateral ischemia may interfere with Ex‐TcPO_2_ results (Abraham et al., 2021), it appears necessary to highly select the patients to be used for a validation study against imaging modalities.

Based on that, we did a retrospective study that includes highly selected patients with PAD, in order to perform an external validation of Ex‐TcPO₂ results at the buttock and calf and to perform the first validation of Ex‐TcPO_2_ at the thigh level. This study also aimed to determine the respective diagnostic performance of the minimal DROP (DROPmin) and the DROP value observed at the end of the walking period (DROPend).

## 2 METHODS

### Study design and ethics

This study was conducted retrospectively, and the ethical approval was obtained from the Ethics Committee of the Centre Hospitalier Universitaire (CHU) d′Angers (approval number: 2024‐236).

### 2.1 Participants

Since 1999, a total of 9,684 records of patients complaining of claudication have been referred for walking investigations in the laboratory of the vascular medicine department of the CHU Angers (France). However, the Ex‐TcPO_2_ testing was initially performed using a limited number of probes at that time. The ability to perform bilateral measurements of the chest, buttocks, thighs and calves was only introduced in 2016.

To be included in this retrospective study, participants had to be over 18 years old, having undergone an Ex‐TcPO₂ testing between January 1st 2016 and December 31th 2024, and have had imaging performed within 1 year before or after the Ex‐TcPO₂ assessment. Pregnant women and people unable to express their consent as well as non‐French‐speaking patients and patients who objected to the use of their data for research were not included. Additional exclusion criteria based on specific test results are detailed in the following sections.

### 2.2 Ex‐TcPO₂ testing and extraction

#### 2.2.1 Ex‐TcPO₂ walking protocol

The Ex‐TcPO₂ testing has been extensively described in previous studies (Abraham et al., 2003; Abraham et al., 2018; Audonnet et al., 2017). In general, Ex‐TcPO₂ is a measurement tool that assesses the oxygen level at the skin surface and indicates the capacity of local circulation to oxygenate the skin tissue. The Ex‐TcPO₂ testing is conducted using calibrated oximetry probes (Perimed; Stockholm, Sweden) with eight electrodes placed on both sides of the body, at the level of the scapulas (for control measurements), as well as on the buttocks, quadriceps, and gastrocnemius muscles while the patient is walking on a treadmill following a standardized protocol (see Supplementary file 1). Each electrode is first set to a temperature of 44.5°C to ensure vasodilation of the vessels and to decrease the oxygen pressure gradient at the skin surface. Then, the measuring device adjusted the temperature of the electrodes to 37°C. Before starting the testing, a calibration period of about 10 min is needed.

The Ex‐TcPO₂ walking protocol begins with a 2‐min transcutaneous oximetry (TcPO₂) measurement while the participant is at rest in a standing position on the Zebris treadmill (Zebris FDM‐T, Germany, 2011) to establish baseline values. The walking protocol then starts with the treadmill set at a 10% incline. The speed increases progressively from 1 km/h to 3.2 km/h and that final speed is maintained for up to 12 min. The walking protocol is stopped at the patient′s request or, if necessary, in the absence of intermittent claudication after a total walking time of 15 min. Following the walking protocol, a 10‐min recovery period in a standing position is implemented to allow the Ex‐TcPO₂ measures to return to their initial values.

#### 2.2.2 DROP index calculation

The DROP index calculation is made using the change in TcPO₂ relative to the initial resting value for each electrode and is adjusted based on the absolute change in TcPO₂ at the scapulas' level. Two different types of DROP are calculated: the final DROP value which represents the value at the moment the walking test is stopped (DROPend), and the lowest DROP value which represents the lowest value observed during the entire walking protocol, including the recovery phase (DROPmin). The presence of ischemia was previously proposed as a DROPmin value below −15 mmHg (Abraham et al., 2018). It is important to note that Ex‐TcPO₂ testing is conducted with electrocardiogram monitoring and under medical supervision.

#### 2.2.3 Data extraction from Ex‐TcPO₂ testing

Data extraction regarding the Ex‐TcPO₂ testing includes absolute resting TcPO_2_ values at each location (chest, buttock, thigh and calf) as well as DROPend and DROPmin. The two DROP values (DROPend and DROPmin) were extracted from both left and right lower limbs for every patient. The ischemic lower limb was determined by the presence of a DROPmin ≤ 15 mmHg in at lease one location (buttock, thigh or calf) and the control lower limb was determined by the absence of a DROPmin ≤ 15 mmHg. In addition, the number of locations with DROPmin ≤ 15mmHG in the identified ischemic lower limb and total walking distance were extracted from Ex‐TcPO_2_ testing available data.

#### 2.2.4 Exclusion criteria based on Ex‐TcPO2 testing

Patients were excluded if they did not have Ex‐TcPO₂ data for all locations (thorax, buttocks, thighs, and calves), were able to walk over 15 min without limitations or did not experience symptoms during the Ex‐TcPO₂ assessment. Patient that had cardiac limitations defined by the reach of their 90% theoretical heart rate during the test, had low hemoglobin levels ( < 9 g) and exhibited hypoxemia during Ex‐TcPO₂ testing were also excluded. These conditions are known to cause non‐vascular exercise limitation, typically associated with normal TcPO₂ values, which could misleadingly suggest low test sensitivity and compromise the validity of the diagnostic analysis. In addition, patients with bilateral ischemia according to the Ex‐TcPO₂ testing were excluded to allow each participant to serve as their own internal control. Patients who did not perform the standard Ex‐TcPO₂ testing, or stopped walking due to factors other than pain related to vascular claudication (e.g., dyspnea) were also excluded.

### 2.3 Data extraction from imaging

All medical records of patients who underwent an Ex‐TcPO₂ testing at the University Hospital Center of Angers and had medical imaging were consulted. The presence of stenosis in the common iliac, external iliac, internal iliac, common femoral, deep femoral, superficial femoral, popliteal arteries, as well as distal arteries such as the anterior tibial artery, the posterior tibial artery and the fibular artery, were extracted from all medical records from both ischemic lower limb and control limb.

For data extraction and coding, the arteries were divided into subgroups as follows: 1) common iliac and common iliac arteries, 2) internal iliac artery, 3) external iliac and common femoral arteries, 4) deep femoral artery, 5) superficial femoral artery and popliteal artery, and 6) distal leg arteries (anterior tibial, posterior tibial, and fibular arteries). The presence of stenosis for all location, excepted distal leg arteries, were encoded as follows: 0 = absence of stenosis, 1 = significant stenosis, layered plaque or 40%‐50% stenosis and 2 = occlusion, subocclusion, pre‐occlusion, severe or very severe stenosis or stenosis equal to or greater than 70%. Regarding distal leg arteries, if there was an occlusion of at least one distal leg artery, a score of 1 was attributed. In absence of distal leg artery occlusion, a score of zero was attributed.

#### 2.3.1 Exclusion criteria based on imaging

Patients with Ex‐TcPO_2_ data that met the inclusion and exclusion criteria but that did not have imaging were excluded. Patients were also excluded if they had undergone MRA instead of CTA.

### 2.4 Other information extracted from medical records

In addition to information related to Ex‐TcPO₂ testing and imaging, patient characteristics such as age, height, weight, sex, medication use, presence of diabetes, smoking status (never smoked, former smoker, or current smoker) and ABI measurement for the ischemic and the control leg were extracted from medical records.

### 2.5 Statistical analyses

Means and standard deviations (SD) were used for continuous variables while percentages were used for reporting proportions. T‐test analyses were conducted for resting TcPO_2_ value at each level (buttock, thigh and calf) between ischemic (*n* = 192) and control (*n* = 192) lower limbs. Pearson correlation analysis was conducted between DROPmin values at each location of the ischemic lower limb and walking time.

Receiver operating characteristic (ROC) curve analyses were conducted to assess the diagnostic performance of the Ex‐TcPO₂ in relation to the binary classification (presence or absence of a stenosis) obtained by imaging for each location (buttock, thigh, calf). More precisely, for each ROC analysis, DROPmin values obtained from the Ex‐TcPO_2_ testing for both ischemic (*n* = 192) and control (*n* = 192) lower limbs were compared to the binary classification obtained from the imaging. This was conducted independently for the three following locations: buttock, thigh and calf. This methodological choice was made to reduce inter‐individual variability and enhance the robustness of the comparative analysis. For each location, the area under the curve (AUC) was calculated to quantify overall accuracy. According to commonly accepted thresholds, an AUC between 0.90 and 1.00 reflects excellent diagnostic accuracy; between 0.80 and 0.89, it indicates good diagnostic accuracy; between 0.70 and 0.79, fair accuracy; and between 0.60 and 0.69, poor accuracy. An AUC between 0.50 and 0.59 is considered failed, while a value below 0.50 suggests no discriminative capacity (Çorbacıoğlu Ş & Aksel, 2023). In addition, sensitivity, specificity, and optimal cut‐off values were determined using the Youden index. The positive predicted value (PPV) and the negative predicted value (NPV) were also calculated for each location. Regarding Ex‐TcPO₂ data, DROPmin and DROPend at each location were used. As imaging techniques such as CTA is considered reference tests for confirming the location of arterial lesions in patients with PAD (Brouwers et al., 2022), the presence (1) or the absence (0) of stenosis, as identified in the patients' medical records using imaging, was used as the diagnosis criterion for each lower limb location (buttock, thigh and calf). First, ROC curve analyses were performed using the presence of a stenosis at each lower limb location, defined as a stenosis of ≥70% (score coded as 2 only). Then, ROC curves were regenerate using a stenosis ≥ 40‐50% (score coded as 1 and 2). A stenosis in the buttock was identified by the presence of at least one occlusion in the common iliac, external iliac, or internal iliac artery. A stenosis in the thigh was defined by the presence of at least one occlusion in the common iliac, external iliac, common femoral, or deep femoral artery. A stenosis in the calf was determined by the presence of at least one occlusion in the common iliac, external iliac, common femoral, superficial femoral, popliteal, or distal leg arteries.

All statistical analyses were conducted using IBM SPSS Statistics version 29 (Armonk, NY: IBM Corp.), with a significance level set at a *p*‐value of <0.050.

## 3 RESULTS

**Figure 1:**
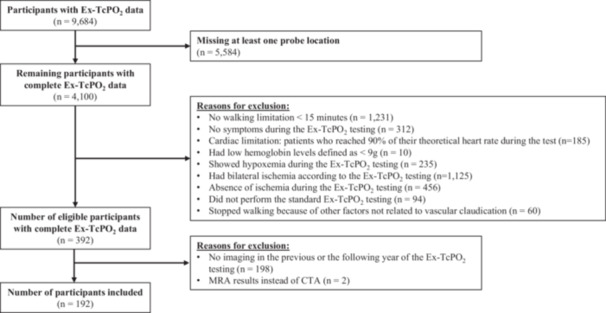
Participants Selection Flowchart.

Among the 9,684 participants with Ex‐TcPO₂ data available in their medical records, only 192 met all inclusion criteria (see Figure 1). Table 1 provides an overview of the included participants' characteristics. Overall, 71.88% of participants were males. The mean resting TcPO₂ values at the chest, as well as the minimum and maximum values, were 64.34 ± 10.31 mmHg, 61.01 ± 10.37 mmHg, and 80.32 ± 11.23 mmHg, respectively. Absolute resting TcPO_2_ values and DROPmin values by location (buttock, thigh and calf) are presented in Supplementary file 2. Absolute resting TcPO_2_ values were respectively 66.51 ± 12.67 mmHg, 68.77 ± 13.54 mmHg and 68.55 ± 11.50 mmHg at the buttock, thigh and calf level for the ischemic lower limbs and were respectively 65.77 ± 11.97 mmHg, 70.44 ± 12.84 mmHg and 68.03 ± 12.53 mmHg at the buttock, thigh and calf level for the control lower limbs. The results of the resting TcPO₂ comparisons between the ischemic and the control lower limbs indicate that there was no significant difference at the buttock (*p* = 0.557), thigh (*p* = 0.215) and calf (*p* = 0.672) levels. Results showed non‐significant correlations between the DROPmin value at buttock (r = −0.102, *p* = 0.161), thigh (r = 0.024, *p* = 0.740) and calf (r = 0.004, *p* = 0.955) location of the ischemic lower limb and the walking time during the Ex‐TcPO_2_ testing. Regarding the presence of significant stenosis on CTA despite a normal Ex‐TcPO_2_ testing on the control limb, significant stenosis was found in 23.9% patients at the buttock, 22.9% patients at the thigh, and 7.4% patients at the calf.

**Table 1:** Participants' characteristics.

Variables	Included participants (*n* = 192)
***Sociodemographic data***	
M: F ratio	138: 54
Age (years), mean ± sd	64.07 ± 10.10
Height (m), mean ± sd	1.68 ± 0.08
Weight (kg), mean ± sd	77.4 ± 15.7
BMI (kg/m^2^), mean ± sd	27.5 ± 5.1
***Health‐related outcomes***	
*Medication intake*	
Antiplatelet agent, *n* (%)	174 (90.6)
Antihypertensive, *n* (%)	125 (65.1)
Hypolipidemic, *n* (%)	147 (76.6)
Anticoagulant, *n* (%)	15 (7.8)
Beta‐blocker, *n* (%)	61 (31.8)
Antidiabetics (oral or insulin), *n* (%)	54 (28.1)
*Smokers*	
Never, *n* (%)	26 (13.5)
Former smoker, *n* (%)	71 (37.0)
Active smoker, *n* (%)	95 (49.5)
*Side of the ischemia according to Ex‐TcPO₂*	
Right, *n* (%)	92 (47.9)
Left, *n* (%)	100 (52.1)
*Number of ischemia by location according to Ex‐TcPO₂ for the ischemic limb*	
Buttock, *n* (%)	66 (34.4)
Thigh, *n* (%)	78 (40.6)
Calves, *n* (%)	176 (81.7)
*ABI measurement*	
Ischemic lower limb, mean ± sd	0.71 ± 0.24
Control lower limb, mean ± sd	0.93 ± 0.24

*Note*: M = males, F = females, BMI = Body Mass Index, Ex‐TcPO₂ = Exercise transcutaneous oximetry.

### 3.1 Performance of Ex‐TcPO₂ compared to imaging

**Figure 2:**
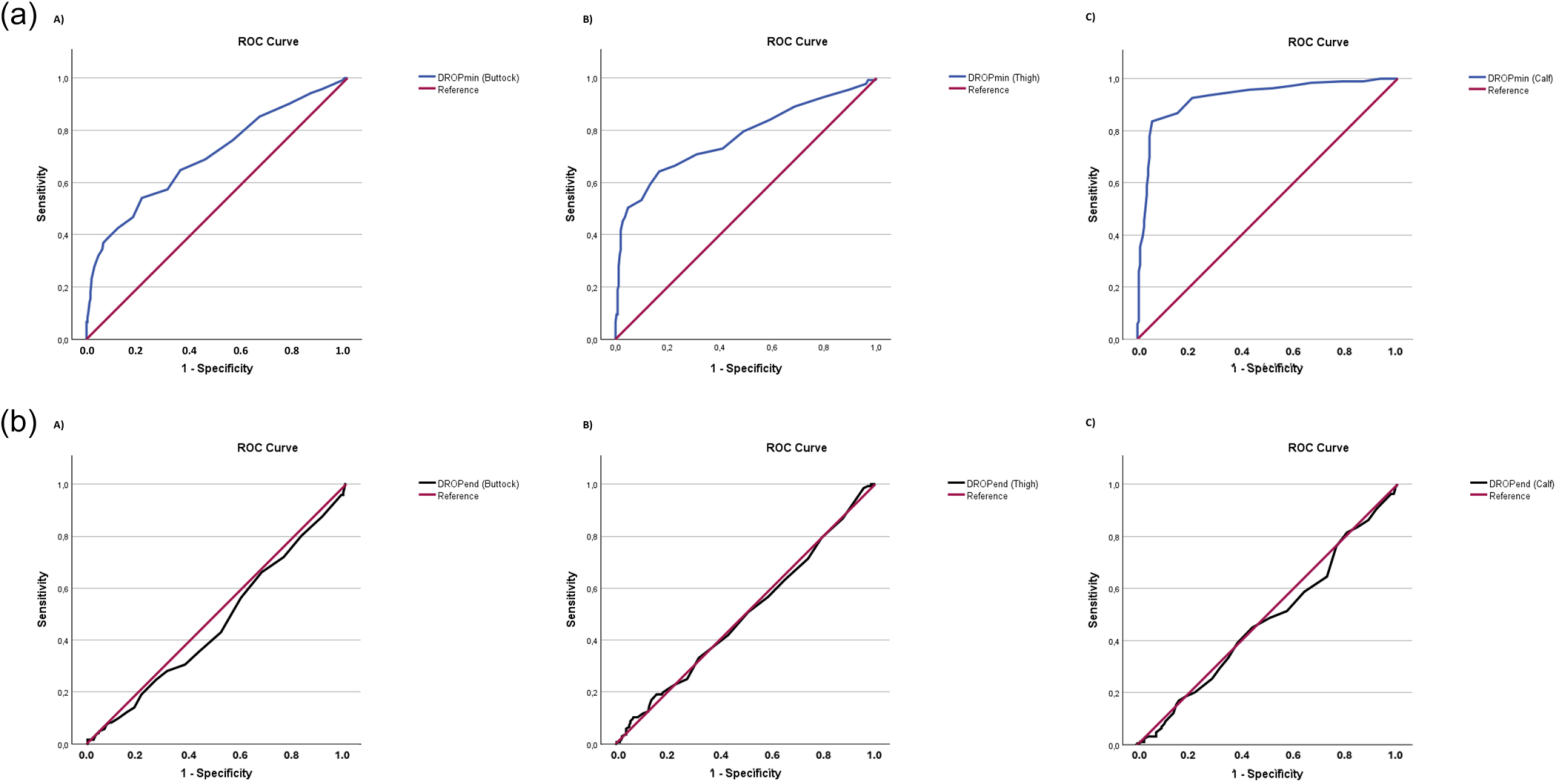
a) ROC curves using DROPmin for (A) the buttock, (B) the thigh, and (C) the calf areas and b) ROC curves using DROPend for (A) the buttock, (B) the thigh, and (C) the calf areas.

The ROC curves associated with the performance of Ex‐TcPO₂ at each lower limb location (buttock, thigh, and calf), using either DROPmin or DROPend, in comparison with imaging indicating a stenosis of ≥70%, are presented in Figure 2.

At the buttock level, ROC curve analysis of the diagnostic performance of Ex‐TcPO₂ using DROPmin showed an AUC of 0.70 (95% CI: 0.64–0.76), indicating fair discriminative ability. The optimal cutoff value, determined using Youden′s index (0.306), was −15 mmHg, with a sensitivity of 38.5% and a specificity of 92.0%. The PPV and NPV were respectively 69.1% and 76.4%. In contrast, when using DROPend, the AUC was 0.46 (95% CI: 0.40–0.53), indicating no discriminative value.

At the thigh level, Ex‐TcPO₂ using DROPmin yielded an AUC of 0.77 (95% CI: 0.72–0.83), indicating fair discriminative ability. The optimal cutoff value, determined using Youden′s index (0.456), was −15 mmHg, with a sensitivity of 50.4% and a specificity of 95.2%. The PPV and NPV were respectively 85.2% and 77.9%. When using DROPend, the AUC decreased to 0.50 (95% CI: 0.44–0.56), indicating no discriminative value.

Finally, at the calf level, Ex‐TcPO₂ using DROPmin showed an excellent discriminative ability, with an AUC of 0.93 (95% CI: 0.90–0.96). The optimal cutoff value, based on Youden′s index (0.780), was −16 mmHg, with a sensitivity of 83.6% and a specificity of 94.4%. The PPV and NPV were respectively 93.5% and 85.6%. When DROPend was used, the AUC decreased to 0.48 (95% CI: 0.42–0.54), indicating no discriminative value.

**Figure 3:**
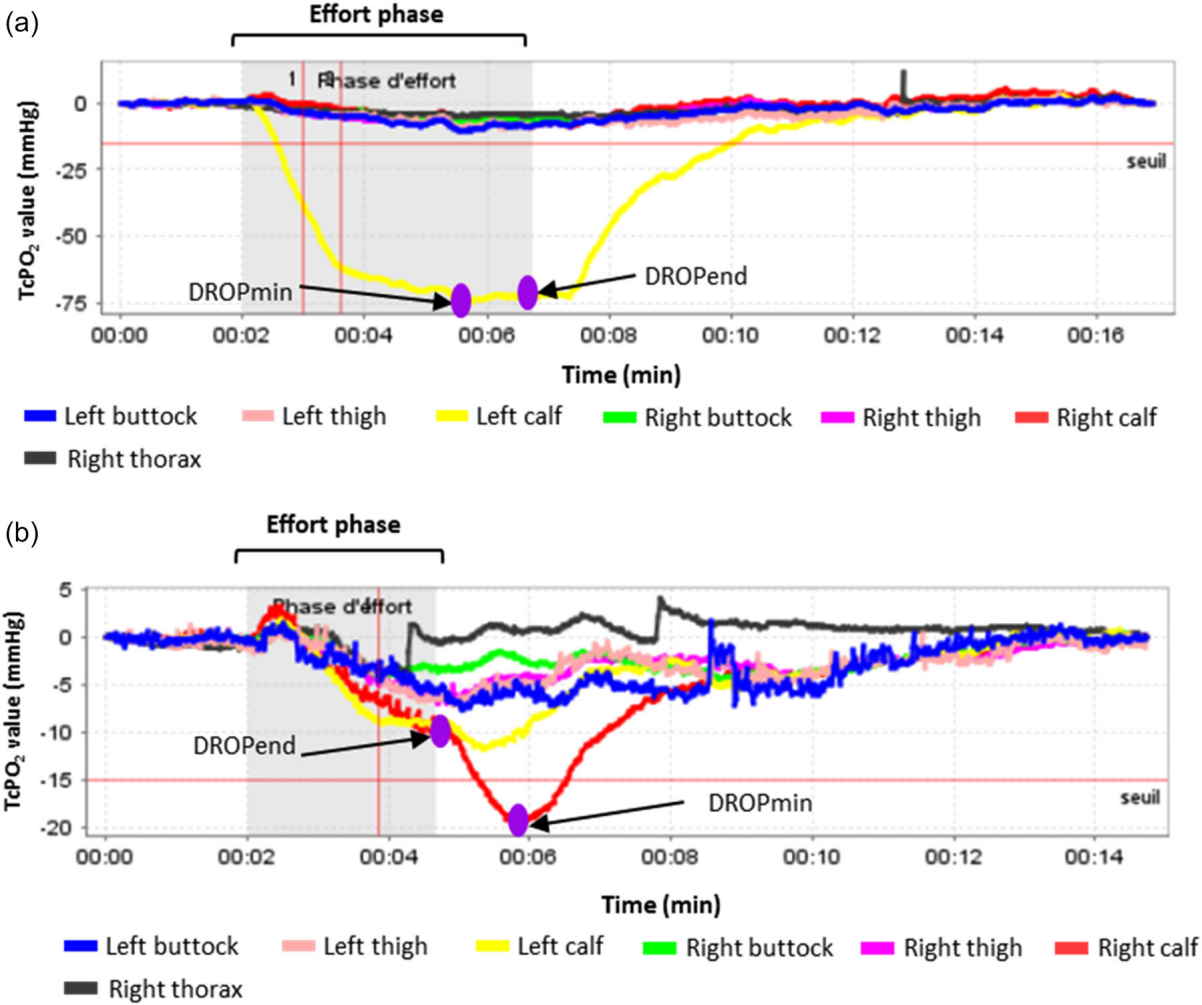
Representative Ex‐TcPO₂ curves illustrating two possible cases, with DROPmin and DROPend indicated. The positions of DROPmin and DROPend can vary across cases, highlighting inter‐subject variability: a) According to both DROPmin and DROPend value, there was a significant ischemia in the left calf of the participant (yellow line). The DROPmin value was obtained during the effort phase (during walking) of the TcPO_2_ recording. Interestingly, both DROPmin and DROPend values for the left calf were similar; b) When looking at the right calf (red line), there was no significant DROPend value. However, the red line continued to decrease during the post‐effort recording allowing us to obtain a significant DROPmin in the post‐effort period.

The performance of Ex‐TcPO₂ at each lower limb location (buttock, thigh, and calf), using DROPmin, in comparison with imaging indicating a significant stenosis ( ≥ 50%) is presented in Supplementary file 3. In addition, the Figure 3 showed examples of recorded Ex‐TcPO_2_ data in patients with suspicion of PAD.

### 3.2 DROPmin values obtained with Ex‐TcPO₂ testing and number of stenosis (≥70%) according to imaging

**Figure 4:**
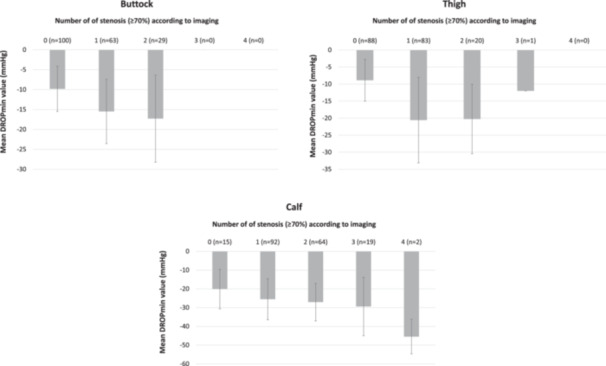
Mean DROPmin values according to number of stenosis ( ≥ 70%) for each location (buttock, thigh and calf).

The Figure 4 showed that, in the limb with at least one ischemic site identified by Ex‐TcPO₂ testing, the mean DROPmin value progressively decreased as the number of stenoses increased, regardless of the location of ischemia (buttock, thigh, or leg).

## 4 DISCUSSION

The aim of the present study was to determine the diagnostic performance of Ex‐TcPO₂ in comparison to imaging for the identification of arterial stenosis in the buttock, thigh or calf area in patients with suspected PAD. Our results showed that Ex‐TcPO₂ had a fair accuracy for detecting at least one stenosis ( ≥ 70%) in the buttock area, a good accuracy for detecting at least stenosis ( ≥ 70%) in the thigh and an excellent accuracy for detecting at least one stenosis ( ≥ 70%) in the calf area, when using the DROPmin value compared to imaging. Furthermore, our findings support the use of a −15 mmHg threshold for the DROPmin value as an optimal cut‐off for Ex‐TcPO₂ testing for the buttock and the thigh locations and the use of a −16 mmHg threshold for the DROPmin value as an optimal cut‐off for Ex‐TcPO₂ testing for the leg. In addition, our results showed that the DROPmin value should be used instead of the DROPend value for the diagnosis of PAD.

The diagnostic accuracy observed in our study aligns with previous findings, reinforcing the utility of Ex‐TcPO₂ in assessing lower limb ischemia. In fact, a prior study evaluating the accuracy of Ex‐TcPO₂ compared to CTA for detecting arterial stenosis in both proximal and distal lower limb levels found that the Ex‐TcPO₂ technique was especially accurate in identifying stenoses ≥ 60% at both sites (Koch et al., 2016). A direct comparison of diagnostic accuracy between our study and that of Koch et al. is not possible, as their model included analysis of the abdominal aorta, whereas ours did not. Generally, an occlusion of the abdominal aorta can significantly impair blood flow to both lower limbs, potentially resulting in bilateral ischemia during walking due to reduced oxygen delivery (Clair & Beach, 2015). As our study focused on unilateral ischemia according to the Ex‐TcPO_2_ testing, only patients with unilateral ischemia observed during the treadmill walking tests were included. However, similarly to our results, Koch and colleagues reported optimal DROPmin cut‐off values of –15 mmHg for the proximal level and –16 mmHg for the distal level, which are consistent with our findings of a DROPmin cut‐off of –15 mmHg at the buttock and thigh levels, and –16 mmHg at the calf level (Koch et al., 2016). Other previous studies also reported an optimal DROPmin cut‐off value of −15 mmHg for the diagnosis of stenosis >75%. Indeed, Audonnet and colleagues reported that cut‐off value of −15 mmHg for both normal and abnormal chest‐TcPO₂ profiles (Audonnet et al., 2017) while Abraham and colleagues first reported the optimal DROPmin cut‐off value being −15 mmHg for proximal ischemia when comparing performance of TcPO_2_ and results from angiography (Abraham et al., 2003). Overall, this highlights the clinical relevance of Ex‐TcPO₂ as a non‐invasive diagnostic tool for PAD and confirm the optimal DROPmin cut‐off value.

According to our results, the sensitivity of Ex‐TcPO_2_ testing for identifying arterial stenosis in the buttock, thigh or calf area in patients with PAD using the DROPmin value was low. This is not surprising since imaging served as a comparator test and a positive result at imaging does not necessarily translate into exercise‐induced ischemia. This could also be explained by the fact that all participants included in our analysis already had a suspected or confirmed diagnosis of PAD before Ex‐TcPO_2_ testing. Indeed, the lack of a true negative group (healthy controls) could have contributed to the lower sensitivity observed in our analysis. In that context, a high sensitivity could not be attempted as the objective was not to use Ex‐TcPO_2_ for screening purposes, but for diagnostic confirmation (Trevethan, 2017). In contrast, the Ex‐TcPO_2_ showed high specificity for all lower limb locations using the DROPmin value. This suggests that the Ex‐TcPO_2_ testing can be considered as a useful complementary tool for the diagnosis of PAD, especially with patients presenting with atypical symptoms. Furthermore, the Ex‐TcPO2 testing may help distinguish vascular claudication present in patients with PAD from other potential causes of exertional lower limb pain such as neurogenic claudication. However, additional studies are still needed to assess its discriminative capacity between vascular and non‐vascular etiologies such as neurogenic claudication.

Regarding the use of DROP values, our results showed that the use of the DROPmin value was more accurate than the DROPend value for identifying stenoses across all locations. The DROPmin value can be obtained either during the walking test or during the 10‐min recovery period following the test. During walking on the treadmill, collateral networks may temporarily compensate for arterial stenosis (Norgren et al., 2007), potentially masking its hemodynamic impact. The development of collateral arteries to improve the blood supply to the lower part of the limbs is often observed in patients with PAD (Golledge, 2022). In this context, there may be no significant DROP during walking on the treadmill despite the onset of symptoms such as cramp and pain because of the presence of developed collateral arteries (Aronow, 2007). However, upon return to rest, blood flow may preferentially redirect through the primary arterial pathway, unmasking the pressure DROP more clearly. This finding highlights the clinical importance of measuring TcPO₂ during at least 10 min following the walking test. This appears of major importance, because, due to the duration of the test, there is a tendency to stop recording and disconnect the probes as soon as the walking period ends. However, this practice is not recommended, as DROPend fails to detect abnormal responses, as shown in our study.

To our knowledge, this is the first study to exclude participants that can present other reasons why oxygen at the chest level may be reduced during exercises and then impact negatively the DROPmin values into the lower limbs. In fact, our study excludes participants with conditions considered as potential cofounding factors. For example, it was decided to exclude patients with low hemoglobin levels or exercise‐induced hypoxemia. The presence of low hemoglobin levels can impact the systemic blood oxygen content. Since more oxygen content is needed during exercise such as walking compared to rest, the decrease of blood flow oxygen content during exercise testing can contribute to low physical performance (Penninx et al., 2004; Steinmeyer et al., 2020) and mobility difficulties in older adults (Chaves et al., 2002; Hirani et al., 2016). In PAD patients, this may lead to a decrease in walking distance that is not only attributable to the presence of arterial stenosis. Regarding exercise‐induced hypoxemia, it has been previously mentioned, a decrease in absolute systemic TcPO₂ values, as measured by chest probes, may reflect subcutaneous oxygen imbalances during exercise (Abraham et al., 2021). This could lead to a misinterpretation of DROPmin values and result in the incorrect attribution of walking limitations to non‐vascular causes of intermittent claudication. For example, if the decrease of the TcPO_2_ in the left thigh mimic the decrease in systemic TcPO_2_ estimated using the chest probe reference in patients with hypoxemia, the DROP values may remain within normal limits. Additionally, the presence of exercise‐induced hypoxemia may impair walking capacity in patients with moderate PAD, due to suboptimal blood flow directly reducing the oxygen content delivered to lower limb muscles during exercise.

### 4.1 Strengths and limitations

A key strength of this study is that it is the first to compare Ex‐TcPO₂ with imaging‐based diagnosis across all arterial territories of the lower limbs, providing a comprehensive evaluation of its diagnostic performance. To our knowledge, this is the first study to assess the diagnostic performance of TcPO₂ using both DROPmin and DROPend values. As the DROPmin showed a better accuracy for all lower limbs arterial territories, the findings of this study support its use as the preferred clinical parameter for interpreting Ex‐TcPO₂ testing results in patients with PAD. Our study is also the first one, to our knowledge, to show the importance of lesions numbers on regional ischemia severity.

This study is, however, not without limitations. The first limitation of this retrospective study is the potential selection bias related to the inclusion of only those patients who underwent further diagnostic investigations based on the result obtained by Ex‐TcPO₂. Consequently, the diagnostic performance of the Ex‐TcPO_2_ test may be overestimated, as patients with negative or inconclusive initial results were not consistently assessed using imaging. More precisely, patients with normal ABI and then normal DROPmin values obtained by the Ex‐TcPO_2_ testing are typically not referred for imaging. In addition, patients who report exertional leg pain but present normal DROPmin values during Ex‐TcPO₂ testing often have borderline or normal ABI with comorbidities such as diabetes or atypical claudication symptoms that can be related to other conditions such as lumbar spinal stenosis, osteroarthritis or mechanical pain.

While the absence of healthy control participants may be considered a limitation in the present study, as it potentially affects estimation of sensitivity of Ex‐TcPO_2_ testing, it aligns with the intended use of the technique. The use of Ex‐TcPO_2_ is not meant for the general population screening, but rather for targeted evaluation of individuals presenting symptoms associated with vascular claudication or atypical lower limb symptoms during walking. In this context, achieving high diagnostic specificity values for all location is considered as more clinically relevant. However, it is important to note that, to minimize bias, inclusion was restricted to patients with unilateral exercise‐induced ischemia, with the contralateral limb serving as an internal control. Overall, our study population consists of highly selected patients with suspected PAD, exercise limitation, unilateral ischemia, and absence of comorbidities that could interfere with walking ability, which limits the generalizability of our findings to broader clinical settings.

### 4.2 Clinical implications

Actually, the ABI measurement remains the initial clinical test most commonly used to screen for the presence of PAD in patients complaining of lower limb pain and walking limitations (Nordanstig et al., 2024). However, ABI is a resting evaluation, and even when resting values are normal, post‐exercise ABI may be performed to confirm or rule out PAD, particularly in patients with exertional limb pain and suspected proximal or segmental arterial lesions. Nonetheless, both resting and post‐exercise ABI are limited in their applicability in cases of non‐compressible arteries or when measurements are technically unfeasible. Moreover, post‐exercise ABI may remain within normal limits in the presence of efficient collateral circulation, potentially masking regional ischemia. Prior research has shown that post‐exercise ABI and Ex‐TcPO₂ are not interchangeable in the assessment of suspected PAD (Mahé et al., 2020). Our findings support the clinical relevance of Ex‐TcPO₂ as a complementary tool in this diagnostic pathway. Ex‐TcPO₂ offers a dynamic and regional assessment of perfusion during effort, enabling detection of both localized and systemic ischemia even in cases of patients where ABI is preserved by collateral flow. Its ability to identify perfusion deficits at the buttock, thigh, and calf levels provides valuable anatomical insight that ABI cannot offer. Moreover, the continuous recording of TcPO₂ during exercise allows for real‐time identification of DROPmin, which is not accessible with post‐exercise ABI. These features make Ex‐TcPO₂ particularly valuable in complex clinical scenarios and may enhance diagnostic accuracy, reduce false negatives, and optimize patient management.

## 5 CONCLUSION

This study showed that Ex‐TcPO₂ as a fair to excellent accuracy with very high specificity (>90%) for detecting the presence of at least one artery stenosis ≥ 70% in the lower limb according to buttock, thigh and calf area in highly selected patients. This study also highlights the importance of using the minimal value of DROP (DROPmin) rather than the DROP value at the end of the walking part of the evaluation (DROPend) values to assessed patients referred to dynamic investigation related to PAD diagnosis. Overall, Ex‐TcPO₂ appears to be a clinically relevant tool for the assessment of PAD, particularly due to its ability to capture exercise‐induced ischemia. Its functional approach may offer complementary diagnostic value in the clinical context of vascular claudication investigation.

## AUTHOR CONTRIBUTIONS

Conception and study design: P.A., M.H., S.L.; Data acquisition: M.H., S.L., M.F., J.H., S.H.; Data extraction: M.H., S.L.; Data analysis: M.H., P.A.; Manuscript writing: M.H., S.L., P.A. All authors have read and approved the final version of this manuscript and agree to be accountable for all aspects of the work in ensuring that questions related to the accuracy or integrity of any part of the work are appropriately investigated and resolved. All persons designated as authors qualify for authorship, and all those who qualify for authorship are listed.

## CONFLICT OF INTEREST STATEMENT

P.A. has benefited from loaning devices from Radiometer, Perimed and Medicap companies in the past. The companies had no access to any of the present manuscript part, concept writing, or discussion.

## Supporting information

Supporting File 1

Supporting File 2

Supporting File 3

## Data Availability

The data that support the findings of this study are available from the corresponding author upon reasonable request.
