# Supplementary material for: Performance of exercise transcutaneous oximetry versus imaging at the buttock, thigh and calf level for the diagnosis of peripheral artery disease
Source: Clin Physiol Funct Imaging. 2026 May 18;46:e70068. doi: 10.1111/cpf.70068 (PMC13184581; doi:10.1111/cpf.70068)
Supplement: Supplementary file 1 — Supporting File 1 [file CPF-46-0-s001.pptx]

## Slide 1
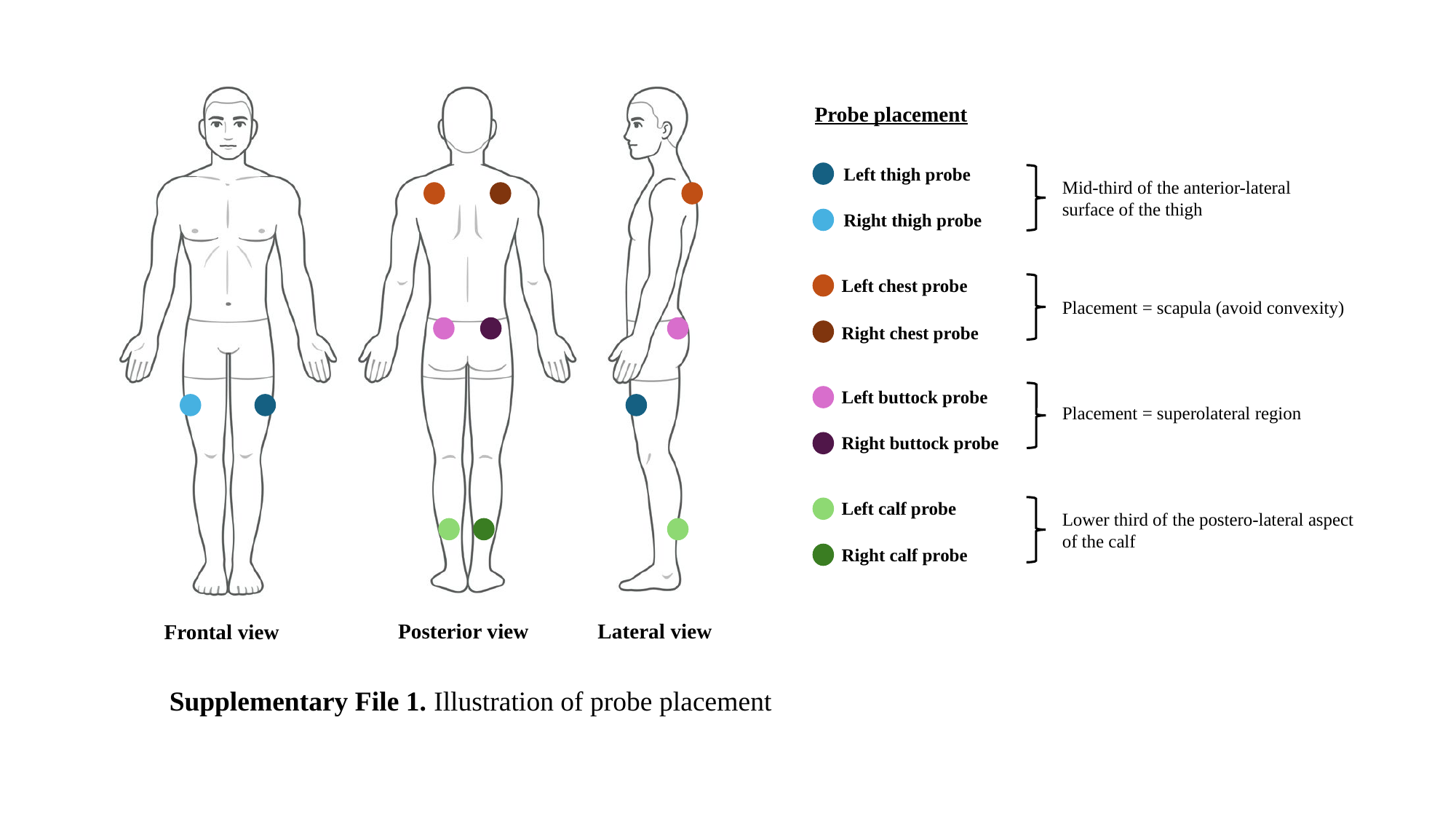

Probe placement
Left thigh probe
Mid-third of the anterior-lateral surface of the thigh
Right thigh probe
Left chest probe
Placement = scapula (avoid convexity)
Right chest probe
Left buttock probe
Placement = superolateral region
Right buttock probe
Left calf probe
Lower third of the postero-lateral aspect of the calf
Right calf probe
Posterior view
Lateral view
Frontal view
Supplementary File 1. Illustration of probe placement
