# Supplementary material for: Performance of exercise transcutaneous oximetry versus imaging at the buttock, thigh and calf level for the diagnosis of peripheral artery disease
Source: Clin Physiol Funct Imaging. 2026 May 18;46:e70068. doi: 10.1111/cpf.70068 (PMC13184581; doi:10.1111/cpf.70068)
Supplement: Supplementary file 2 — Supporting File 2 [file CPF-46-0-s002.pptx]

## Slide 1
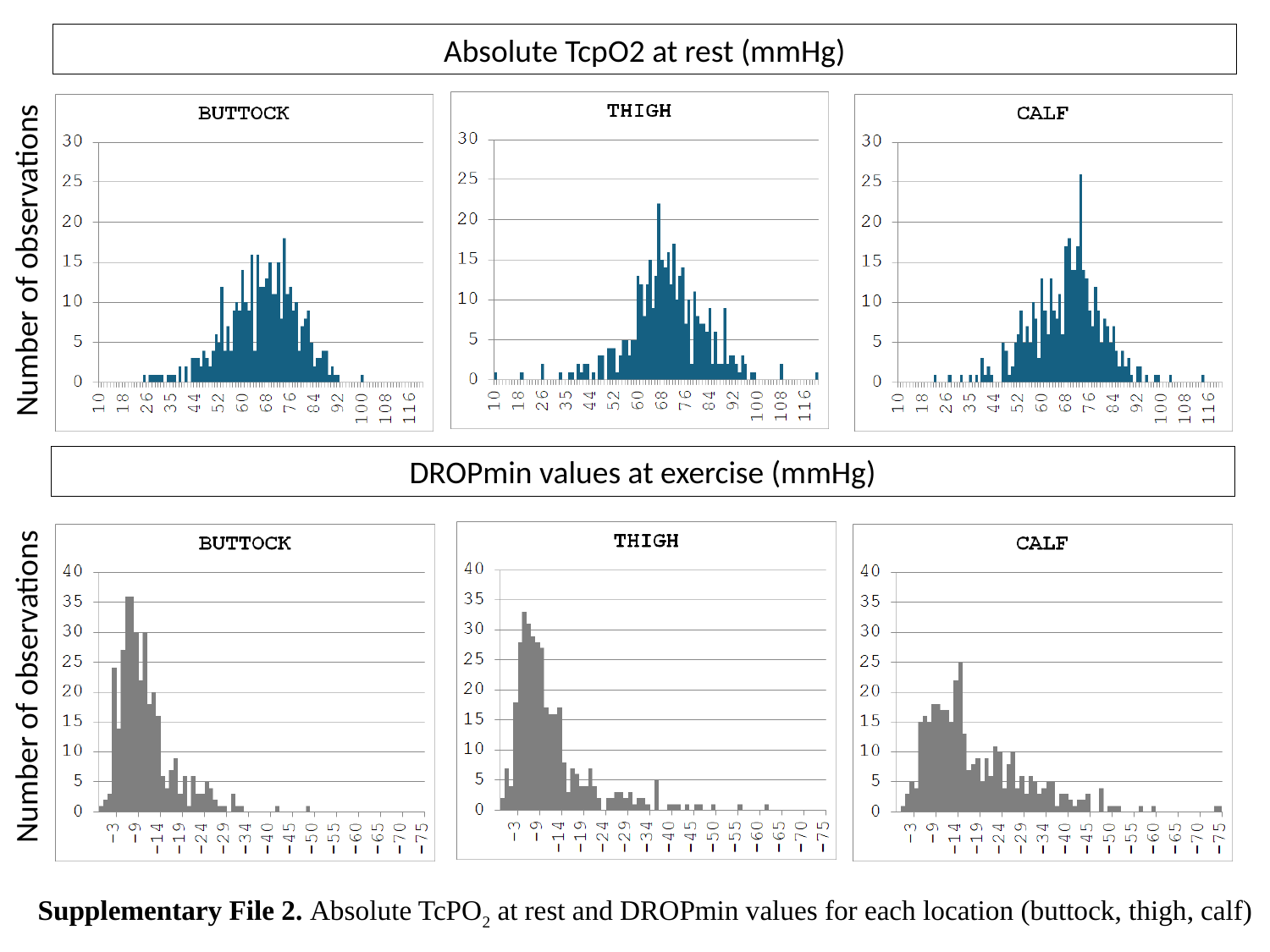

Absolute TcpO2 at rest (mmHg)
Number of observations
DROPmin values at exercise (mmHg)
Number of observations
Supplementary File 2. Absolute TcPO2 at rest and DROPmin values for each location (buttock, thigh, calf)
