# Supplementary material for: Performance of exercise transcutaneous oximetry versus imaging at the buttock, thigh and calf level for the diagnosis of peripheral artery disease
Source: Clin Physiol Funct Imaging. 2026 May 18;46:e70068. doi: 10.1111/cpf.70068 (PMC13184581; doi:10.1111/cpf.70068)
Supplement: Supplementary file 3 — Supporting File 3 [file CPF-46-0-s003.docx]

**Supplementary file 3 – Figure A**

When generating ROC curves using a stenosis threshold of ≥40–50% (scores coded as 1 and 2), the performance of Ex-TcPO₂ at the buttock level using DROPmin values yielded an AUC of 0.69 (95% CI: 0.63–0.74), indicating poor discriminative ability. The optimal cutoff value, determined using Youden’s index (0.284), was −12 mmHg, with a sensitivity of 48.4% and a specificity of 80.0%.

For the thigh level, the performance of Ex-TcPO2 using DROPmin values ROC curves analysis showed AUC of 0.73 (95% CI: 0.68–0.78), indicating fair discriminative ability. The optimal cutoff value, determined using Youden’s index (0.404), was −12 mmHg, with a sensitivity of 55.4% and a specificity of 85.0%.

For the calf level, the performance of Ex-TcPO2 using DROPmin values ROC curves analysis showed AUC of 0.79 (95% CI: 0.75–0.84), indicating fair discriminative ability. The optimal cutoff value, determined using Youden’s index (0.504), was −16 mmHg, with a sensitivity of 54.2% and a specificity of 96.2%.

**
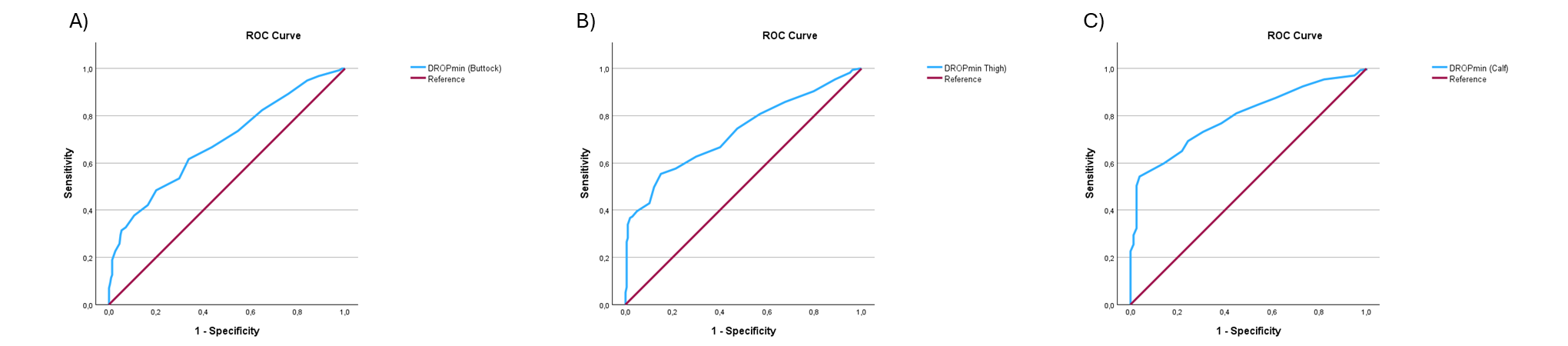
**

**Figure A.** ROC curves using DROPmin for (A) the buttock, (B) the thigh, and (C) the calf areas.
